# The Frequency of Exotoxin A and Exoenzymes S and U Genes Among Clinical Isolates of *Pseudomonas* *aeruginosa* in Shiraz, Iran

**Published:** 2015

**Authors:** Arshid Yousefi-Avarvand, Reza Khashei, Hadi Sedigh Ebrahim-Saraie, Amir Emami, Kamiar Zomorodian, Mohammad Motamedifar

**Affiliations:** 1 *Department of Bacteriology and Virology, School of Medicine, Shiraz University of Medical Sciences, Shiraz, Iran.*; 2 *Department of Parasitology and Mycology, School of Medicine, Shiraz University of Medical Sciences, Shiraz, Iran.*; 3 *Shiraz HIV/AIDS Research Center, Shiraz University of Medical Sciences, Shiraz, Iran.*

**Keywords:** *Pseudomonas aeruginosa*, exotoxin A, exoenzyme S, exoenzyme U

## Abstract

*Pseudomonas aeruginosa *as an opportunistic pathogen produces several virulence factors. The most important of these factors are exotoxin A and type III secretion system (T3SS). The aim of this study was to determine the frequency of *toxA*, *exoU* and *exoS* genes among clinical isolates of* P. aeruginosa*. In this cross-sectional study from September 2011 to February 2012, 156 *P. aeruginosa *isolates were recovered from different clinical samples. Susceptibility testing against 10 antibiotics was performed on individual isolates by the disc diffusion method according to CLSI guidelines. Extracted DNA was subjected to PCR assay for determining the presence of *toxA*, *exoU* and *exoS* genes. Overall, the frequency of *toxA*, *exoU* and *exoS* genes were 90.4%, 66.7% and 65.4%, respectively. All of the abdominal and eye isolates were *exoS*^+^. The frequency of *exoS*^+^/*exoU*^-^ and *exoS*^-^/*exoU*^+^ genotypes was estimated 19.2% and 16.2%, respectively. Indeed, genotypes *exoS*^+^/*exoU*^+^ and *exoS*^-^/*exoU*^-^ were found with frequencies of 48.7% and 15.3%, respectively. The highest and lowest antibiotic resistance rate was seen against azteroenam (94.2%) and amikacin (44.9%), respectively. Fluoroqinolone-resistant isolates were isolated with frequency of 45.8%. Multi-drug resistant (MDR) isolates were detected in 62.8% of isolates. The resistance rate in *exoU*^+^ isolates was 86% compared to 66% in *exoS*^+^ isolates. The high frequencies of virulence genes detected in our clinical isolates with notable antibiotic resistance rates indicate the potential risk of these isolates in nosocomial infections.

*Pseudomonas aeruginosa* is a Gram-negative and opportunistic pathogen which is widesp-read throughout the environment (1). In immune competent hosts, the bacterium seldom causes disease (1, 2). *P. aeruginosa* causes a wide range of infections including septicemia, pneumonia, endo-carditis, burn wounds, otitis and keratitis. It is also frequently encountered in cystic fibrosis, immuno-compromised and/or hospitalized individ-uals (3-5). The pathogenesis of *P. aeruginosa* is multifactorial and comprises several cell-associated and extra-cellular virulence determinants. These include exo-toxin A (ExoA), phospholipase, elastase, pyocya-nin, pili, flagella and lipopolysac-charide (3,6,7). Proliferation of *P. aeruginosa* in host cells and overcoming defense mechanisms is due to the transfer of many proteins via specialized secretion apparatuses including the types I,II,III,V and VI secretary systems (TSS) (2, 8).

ExoA is the major member of the type II secretion system (T2SS) which inhibits protein synthesis by ADP-ribosylation of eukaryotic elongation factor 2 (1, 8, 9). Another important virulence factor recently recognized is the type III secretion system (T3SS) (3, 6). T3SS is a contact-dependent protein secretion pathway that plays a major role in the pathogenesis of serious *P. aeruginosa* infections (10). The four well known T3SS effectory molecules are exoenzymes (Exo) S, U, T and Y (2). It is known that these effectors are delivered to the host cells via a translocation complex consisting of products encoded by the *pcrGVHpopBD* operon that cause cell necrosis and modulation of actin cytoskeleton, allowing the bacteria to invade the eukaryotic cells and escape phagocytosis (3, 6, 10).

ExoS is a major cytotoxin involved in stages of colonization, invasion and dissemination of infection (11). ExoU is a potent cytotoxin with phospholipase activity, capable of killing a variety of eukaryotic cells *in* *vitro* (12, 13). Additionally, ExoU has a greater effect than other T3SS effectors on the virulence of the bacteria (10).

A key determinant of *P. aeruginosa *is its remarkable resistance to antibiotics and notably many of isolates are multidrug-resistant (MDR) (14). In several studies, the relationship between MDR isolates and presence of genes encoding T3SS, especially ExoU has been demonstrated (10, 14).

As the presence of T3SS encoding genes in clinical isolates of *P. aeruginosa *is a variable trait, distribution of these genes in different populations should be explored. Moreover, to the best of the authors' knowledge there is no previous report from Shiraz investigating the prevalence of *P. aeru-ginosa* virulence genes. Therefore, the present study aimed to evaluate the frequencies of *toxA, exoS* and *exoU *genes among the different clinical isolates of *P. aeruginosa*. We also sought to determine whether there is any correlation between the presence of these genes and antibiotic resistance profile of the isolates.

## Materials and methods

**Study design and clinical specimens**

In this cross-sectional study, a total 156 *P. aeruginosa* clinical isolates were obtained from September 2011 to February 2012 in Shiraz (a major city in the south of Iran) teaching hospitals. The samples included: urine, cerebrospinal fluid (CSF), sputum, abdominal discharge (AD), endotr-acheal tube aspirates (ETT), eye, blood, and wound.

**Bacterial identification**

All the isolates were identified as *P. aeruginosa* using both the conventional microbio-logic (e.g., Gram staining, capacity for growth at 42 C, oxidase, and IMViC tests) methods and Microgen ^TM ^GnA+B-ID System (Microgen Bioproducts Ltd, U.K) diagnostic kit. Confirmed *P. aeruginosa* isolates were stored in tryptic soy broth (TSB) (Merck Co., Germany) containing 20% glycerol at -70 C until further study.

**Antibiotic susceptibility testing**

The antimicrobial susceptibility test was done by disk diffusion method on Muller-Hinton agar (Merck Co., Germany). The following antibiotics were tested: Ceftazidime (30 g), Aztreonam (30 g), Gentamicin (10 g), Piperacillin+Tazobactam (110 g), Amikacin (30 g), Ticarcillin (75 g), Ciprofloxacin (5 g), Imipenem (10 g), Ofloxacin (30 g) and Meropenem (10 g) (MAST Co., U.K), in accordance with clinical and laboratory standards institute (CLSI) recommendations (15). MDR isolates were defined if they showed simultaneous resistance to 3 antibiotics. The *P. aeruginosa* ATCC 27853 was used as a reference strain for the quality control of susceptibility test. The intermediate isolates were accounted as resistant in our results.

**Genomic DNA purification and molecular assay**

**Fig. 1:**
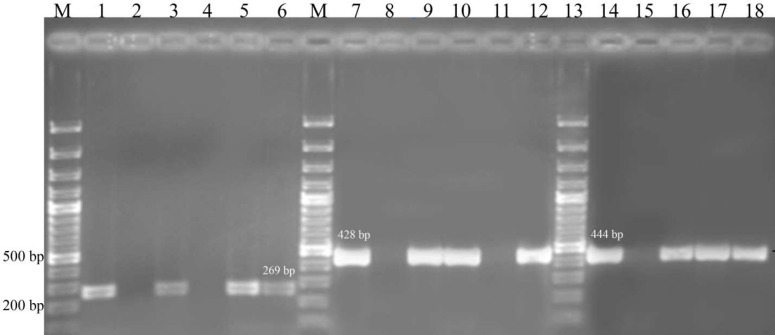
Amplification of* toxA*, *exoS* and *exoU* genes from clinical isolates of *P. aeruginosa* by PCR. M: 100 bp ladder; lane 1: *toxA* gene positive control; lanes 2, 8 and 15: negative control (*E. coli* ATCC 35218); lanes 3-6 investigated clinical isolates for *toxA* gene; lane 7: *exoU* gene positive control; lanes 9-12: investigated clinical isolates for *exoU* gene; lane 14: *exoS* gene positive control; lanes 16-18: investigated clinical isolates for *exoS *gene

Genomic DNA was extracted from overnight TSB cultures of *P. aeruginosa* isolates using the small-scale phenol-chloroform extraction method (16). The evaluation of *toxA*, *exoS* and *exoU* genes was accomplished by previously described primers (9,17). PCR amplification was performed in 50 l reaction volume consisting of 5 l 1x PCR buffer, 2 µl of each primer (10 pmol/µl), 1 l MgCl2 (1.5 mM), 0.8 l each of the dNTPs (200 µM), 0.6 l Taq DNA polymerase (1 U), and 2 l DNA (10-1,000 ng) from each isolate. The PCR cycling conditions were: initial denaturation at 95 °C for 10 min, followed by 30 cycles (for *exoA* gene) and 35 cycles (for *exoU* and *exoS* genes) of denaturation at 95 °C for 30 seconds, annealing at 55 °C for 45 seconds, extension at 72 °C for 40 seconds, with final extension at 72 °C for 10 min. All reagents were obtained from Cinnagene Co., Iran. In each run of amplicons, *P. aeruginosa* ATCC 27853 which is positive for both *toxA* and *exoS*, and one clinical isolate which became positive for the presence of *exo U* gene (428 bp amplicon) and was further confirmed by DNA sequencing, and negative (*E. coli* ATCC 35218) controls were included in agarose gels. The amplicons were resolved in a 1% horizontal agarose gel, stained with ethidium bromide and photographed under 300 nm UV light (Figure 1).

**Statistical analyzes**

Statistical analyses were performed using SPSS^TM^ software, version 21.0. The results for antimicrobial susceptibility and the current genes were presented as descriptive statistics in terms of relative frequencies. Chi–square test or Fisher's exact test was used to analyze the results wherever needed and a p-value< 0.05 was considered as significant clinical relevance.

**Table 1:** Antibiotic susceptibility profile of *P. aeruginosa* isolates based on source of isolation

**Antibiotic** **Specimen**	**CAZ** **No. (%)**	**IMI** **No. (%)**	**OFX** **No. (%)**	**GM** **No. (%)**	**PTZ** **No. (%)**	**TC** **No. (%)**	**MER** **No. (%)**	**CIP** **No. (%)**	**AZT** **No. (%)**	**AK** **No. (%)**
Urine (57)	21 (37)	37 (65)	28 (49)	30 (53)	16 (28)	15 (26)	32 (56)	30 (53)	3 (5.3)	37 (65)
CSF (4)	3 (75)	4 (100)	3 (75)	3 (75)	2 (50)	2 (50)	4 (100)	3 (75)	1 (25)	4 (100)
Sputum (35)	10 (29)	19 (54)	14 (40)	16 (46)	5 (14)	7 (20)	18 (51)	19 (54)	2 (5.7)	16 (46)
Abdominal (4)	1 (25)	3 (75)	1 (25)	3 (75)	0	1 (25)	3 (75)	3 (75)	0	3 (75)
ETT (19)	5 (26)	11 (58)	9 (47)	11 (58)	6 (32)	5 (26)	10 (53)	10 (53)	2 (11)	12 (63)
Eye (5)	2 (40)	1 (20)	1 (20)	2 (40)	1 (20)	0	3 (60)	3 (60)	0	3 (60)
Blood (3)	3 (100)	2 (67)	2 (67)	2 (67)	2 (67)	2 (67)	2 (67)	2 (67)	0	2 (67)
Wound (29)	7 (24)	7 (24)	6 (21)	5 (17)	4 (14)	1 (3.4)	8 (28)	6 (21)	1 (3.4)	9 (67)
Total (156)	52 (33.3)	84 (53.8)	64 (41)	72 (46.2)	36 (23.1)	33 (21.2)	80 (51.3)	76 (42.9)	9 (5.8)	86 (55.1)

CAZ: Ceftazidime; IMI: Imipenem; OFX: Ofloxacin, GM: Gentamycine; PTZ: Piperacillin/ tazobactam; TC: Ticarcillin; MER: Meropenem; CIP: Ciprofloxacin; AZT: Aztreonam; AK: Amikacin

**Table 2:** Distribution of *P. aeruginosa* isolates virulence genes based on source of isolation

**Genes** **Specimen**	***toxA*** No. (%)	***exoS *** b No. (%)	***exoU *** b No. (%)
Urine (57) c,d	49 (86.0)	32 (56.1)	33 (57.9)
CSF (4)	2 (50.0)	3(75.0)	2 (50.0)
Sputum (35)	30 (85.7)	24 (68.6)	23 (65.7)
Abdominal (4)	4 (100.0)	4 (100.0)	2 (50.0)
ETT (19) c,d	19 (100.0)	14 (73.7)	14 (73.7)
Eye (5)	5 (100.0)	5 (100.0)	4 (80.0)
Blood (3)	3 (100.0)	1 (33.3)	2 (66.7)
Wound (29) a,c,d	29 (100.0)	21 (72.4)	22 (75.9)
Total	141 (90.4)	104 (66.7)	102 (65.4)

a Frequency of *toxA* among wound samples was significantly higher than urine isolates (P< 0.05) and distribution of *toxA* among other sources was not significantly different

b Distribution of *exoS* and *exoU* among clinical samples showed no significant differences*.*

c Significant differences of *toxA *frequency compared to *exoS *among individuals clinical samples (P< 0.05)*.*

d Significant differences of *toxA *frequency compared to *exoU *among individuals clinical samples (P< 0.05)*.*

## Results

Of the total 156 *P. aeruginosa *isolates, majority of isolates were recovered from urine (n= 57) and sputum (n= 35) specimens (Table 1). The results of antibiotic susceptibility revealed that tested* P. aeruginosa* isolates were mostly sensitive to amikacin (55.1%), imipenem (53.8%) and meropenem (51.3%). On the other hand, the most resistance rates were seen against aztreonam (94.2%), ticarcillin (78.8%) and piperacillin /tazobactam (76.9%). The results of antibiotic susceptibility profile for *P. aeruginosa* isolates were summarized in Table 1. Moreover, among the 156 isolates, 98 (62.8%) were MDR.

The presence of* toxA*, *exoS* and *exoU* genes was detected in 90.4%, 66.7% and 65.4% of all the isolates, respectively. The distribution of virulence genes among different clinical samples is shown in Table 2. The *toxA* and *exoS* were detected among all the isolates from AD and eye samples. However, the relative frequency of* exoU* was higher in the eye (80%) and wound (75.9%) compared to that seen in other clinical samples. Although, relative frequencies of virulence genes were different between clinical specimens, statistical analysis showed no differences for the presence of individual genes and source of isolation. The only exception was *toxA* which was significantly *(P <0.05)* higher among wound isolates (29/29) compared to urine ones (49/57). Moreover, isolates recovered from urine, wound and ETT samples contained significantly *(P<0.001)* a higher frequ-ency of* toxA* gene than *exoS* and *exoU*. The fre-quency of *exoS*^+^*/exoU*^-^, *exoS*^-^*/exoU*^+^, *exoS*^+^*/exoU*^+^ and *exoS*^-^*/exoU*^- ^genotypes were 19.2%, 16.2%, 48.7% and 15.3%, respectively.

The *exoA*^+^, *exoS*^+^ and *exoU*^+ ^genotypes demonstrated a higher spread (96.9%, 67.3% and 72.4%, respectively) among MDR than non-MDR isolates *(P <0.05).* Indeed, resistance to aztreonam (74.4%) was mainly associated to the isolates harboring *exoA* in comparison to other genes *(P <0.05).*

## Discussion

As detection of virulence genes in clinical isolates of *P. aeruginosa* is important (3,18), thus, in the present study, the frequency of some* P. aeruginosa *virulence genes among different clinical isolates was characterized. As among T3SS, *exoT* and *pcrV *(part of the injection apparatus of the T3SS) genes exist in nearly all *P. aeruginosa* isolates from both clinical and environmental origins (3,12,19), the presence of these genes was not evaluated in this study.

In our investigation, 100% of *P. aeruginosa* isolates from the AD, ET, eye, blood and wound samples were *toxA*^+^*. *The *exoS* gene was also detected in 100% of isolates from the AD and eye samples. According to our results, the relative frequencies of virulence genes were higher in some special clinical specimens. It has been suggested that the infection site and duration of disease influences the virulence of *P. aeruginosa *clinical isolates by altering the production of some virulence determinants. For example, some anatomical sites enhance the production of ExoA and ExoS (10).

In a research in Poland by Wolska et al., the prevalence of *toxA* among the 62 clinical isolates of *P. aeruginosa* was 88.7%, which is comparable with our study. However, the frequency of *exoS* was more than that of the present study (75.8% vs. 66.7%) (17). It seems that the *exoU* had been acquired through a mobile element (plasmid) integrated into the chromosome of *P. aeruginosa*. Therefore, the lower prevalence of the gene than the other virulence genes could be due to this phenomenon (11). However, in our analysis, this rate was nearly the same as *exoS* prevalence (65.4% vs. 66.7%). In the study conducted by Mitove et al. (20) on 202 cystic fibrosis (CF) and non- CF patients, the frequency of *exoS* was 62.4%, whereas the prevalence of *exoU* was found to be 30.2%, which is not in agreement with our study. Interestingly, in a survey in France, the prevalence of *exoS* was markedly higher than other studies (94% in CF isolates vs. 80% in non-CF isolates) (21).

Wareham et al. showed a significant correlation between the distribution of* exoS*^+^/*exoU*^-^ and *exoS*^-^/*exoU*^+^ genotypes with CF (sputum sample) and blood isolates, respectively, which is in concordance with our results (13). Likewise, these results are reported by Feltman et al., who had established an association between *exoS*^+^/*exoU*^+^ genotype with CF isolates (6). In another work by Wong–Beringer et al. exoS^+^/*exoU*^–^ (9%) and *exoS*^-^/*exoU*^-^ (2%) genotypes were found among 45 clinical isolates of *P. aeruginosa* (14). Moreover, in a study conducted on 55 keratitis isolates, 64% of isolates contained* exoS*^+/^*exoU*^–^ genotype, whereas 33% and 4% were *exoS*^-^*/exoU*^+^, and *exoS*^+^*/exoU*^+^, respectively (18).

It is generally suggested that isolates from clinical setting contain either *exoS* or *exoU* gene but not both (22). However, interestingly, in our analysis, 48.7% and 15.3% of the isolates had *exoS*^+^*/exoU*^+^ and *exoS*^-^*/exoU*^-^genotypes, respective-ly. Similar to our survey, in the study of Finnan et al., 75% of isolates were *exoS*^+^*/exoU*^+^ (23).

Our results showed that the overall resistance to azteronam (94.2%) and ticarcillin (78.8%) was remarkably high. Among clinical specimens, AD and eye isolates were 100% resistant to aztreonam. Furthermore, isolates from CSF samples were not resistant to 6 antibiotics of 10 tested antimicrobial agents. But the isolates obtained from other clinical samples showed resistance (with different rates) to all antibiotics. The lowest resistance was seen against amikacin (44.9%), which seems to be the *in vitro* drug of choice in our investigation.

Fluroquinolones such as ciprofloxacin and levofloxacin have a great potency *in vitro* on *P. aeruginosa*; however, because of the widespread usage of these agents, resistance to them has developed (10). In the present study, the overall resistance to ciprofloxacin and ofloxacin were 43.6% and 48.1%, respectively. In contrast to our results, in the study of Choy et al., resistance to fluoroquinolones was found to be 11%. However, this resistance was only related to ofloxacin, whereas all isolates of *P. aeruginosa* were sensitive to ciprofloxacin (18). A relatively high resistance was cited by Agnello et al. with 54% of pneumonic isolates of *P. aeruginosa* being resistant to levofloxacin (10).

On the other hand, in our investigation 62.8% of the resistant isolates exhibited MDR phenotype. The MDR results, is nearly consistent with the finding of a study in Bulgaria with 57.5% prevalence (20). It has been suggested that the clinical isolates comprising *exoU* gene were significantly associated with MDR phenotype (18). This finding is supported in our results, since MDR resistance rate in *exoU*^+ ^isolates with 80% frequency was higher compared to 66% in *exoS*^+^ isolates. The prevalence of such high resistant *P. aeruginosa* in our region is not uncommon, since, previously Anvarinejad et al. and Sarhangi et al. showed high rate of MDR among isolates from the burn patients and clinical isolates from Shiraz City, respectively (24, 25).

One of the limitations of this study was the small sample size of specimens such as blood, CSF, which could affect the results. However, beside the limitations, the current study has some outcomes. First we showed a relatively high frequency of *toxA*, *exoS* and *exoU* genes among *P. aeruginosa* clinical isolates obtained in our area and this frequency was associated to high antibiotic resistance among the isolates. Second, it seems that the source of bacterial isolation is associated with the trend of acquisition of specific virulence genes and these genes may serve to cause specific infections. These results indicate the potential risk of these isolates in nosocomial infections which merit more attention. Of course, further studies are required with larger sample size and from other regions of country to reach a comprehensive conclusion.
